# Single-Cell RNA Sequencing and Combinatorial Approaches for Understanding Heart Biology and Disease

**DOI:** 10.3390/biology13100783

**Published:** 2024-09-30

**Authors:** Le Wang, Bo Jin

**Affiliations:** Department of Clinical Laboratory, Peking University First Hospital, Beijing 100034, China; wangle@fuwai.com

**Keywords:** single-cell RNA sequencing, single-cell multi-omics, heart development and disease

## Abstract

**Simple Summary:**

Scientists have developed new techniques that allow them to study the heart at an incredibly detailed level, focusing on individual cells rather than looking at groups of cells together. This is important because the heart is made up of many different types of cells, each with its own role, and understanding how they interact is key to discovering what happens in both healthy hearts and in heart disease. By using methods that look at the genes, proteins, and other molecules inside single cells, researchers can uncover how cells communicate and change over time. These discoveries are helping scientists learn more about how the heart develops, how it stays healthy, and what goes wrong in heart diseases. This knowledge could lead to better treatments and new ways to diagnose heart conditions, helping people live longer, healthier lives.

**Abstract:**

By directly measuring multiple molecular features in hundreds to millions of single cells, single-cell techniques allow for comprehensive characterization of the diversity of cells in the heart. These single-cell transcriptome and multi-omic studies are transforming our understanding of heart development and disease. Compared with single-dimensional inspections, the combination of transcriptomes with spatial dimensions and other omics can provide a comprehensive understanding of single-cell functions, microenvironment, dynamic processes, and their interrelationships. In this review, we will introduce the latest advances in cardiac health and disease at single-cell resolution; single-cell detection methods that can be used for transcriptome, genome, epigenome, and proteome analysis; single-cell multi-omics; as well as their future application prospects.

## 1. Introduction

The heart is a highly complex organ composed of various cell types, exhibiting not only significant structural complexity but also unique electrical conduction and cellular functionality. This intricate cellular communication and functional heterogeneity present substantial challenges to traditional bulk RNA sequencing. Single-cell RNA sequencing (scRNA-seq) offers high-resolution gene expression analysis and has revolutionized the field of molecular biology. It enables the identification and characterization of distinct cell populations, revealing rare cell types and states that play critical roles in heart function and pathology [1,2]. This technology has opened new avenues for understanding the complex cellular heterogeneity and dynamics within tissues, particularly in cardiac biology and disease [3].

Cardiac development is a highly orchestrated process, originating from multipotent progenitor cells that contribute to the formation of the heart’s diverse cell types. Mesp1, a key regulator of early cardiovascular lineage commitment, marks the earliest known cardiac progenitor population that gives rise to various cardiac lineages [4]. As development proceeds, Nkx2-5, a fundamental cardiac transcription factor, plays a crucial role in further defining cardiac progenitor cells (CPCs), ensuring their proper differentiation and morphogenesis of the heart [5]. A subset of these CPCs, co-expressing Isl1 and Nkx2-5, is essential for the development of specific heart regions, such as the second heart field, which contributes to structures like the right ventricle and outflow tract [6,7]. Understanding these progenitor populations and their markers is vital for deciphering the heterogeneity of cardiomyocytes and their roles in both heart development and disease.

Heterogeneity within cardiac tissues is not limited to cellular identity; it also extends to functional properties and responses to environmental cues. The diverse population of cardiomyocytes, including atrial, ventricular, and pacemaker cells, exhibits distinct electrophysiological characteristics and gene expression profiles, contributing to the heart’s ability to respond to various physiological demands [8]. Moreover, non-cardiomyocyte populations, including fibroblasts, endothelial cells, and immune cells, further contribute to the complexity of the cardiac microenvironment and play essential roles in maintaining homeostasis and responding to injury [9]. The integration of scRNA-seq with spatial transcriptomics and other multi-omic approaches enables researchers to unravel these layers of heterogeneity, offering a comprehensive understanding of cellular interactions and functional dynamics within the heart [10].

Yet of the many types of molecules and biological processes that define the status and activity of an individual cell, mRNA expression represents a single member of the consortium of parameters that together define cellular identity and cell fate. Therefore, merely investigating the transcriptome may provide only a limited snapshot of the intricacies within cells. As such, integrating other levels of assessments, such as that of genomics, epigenomics, proteomics, and metabolomics, is projected to synergize in producing meaningful biological discoveries. Spatial transcriptomics allows for the localization of gene expression within the tissue environment, preserving spatial organization. Similarly, integrating scRNA-seq with single-cell ATAC-seq provides insights into the epigenetic regulation of gene expression at the single-cell level [3]. These multi-omic approaches have already led to significant discoveries in cardiac biology, such as the identification of new regulatory networks and cell interactions contributing to heart disease [11]. Additionally, single-cell proteomics offers a comprehensive analysis of protein expression and modifications at the single-cell level, revealing the relationship between gene expression and protein translation [12,13,14].

**Figure 1:**
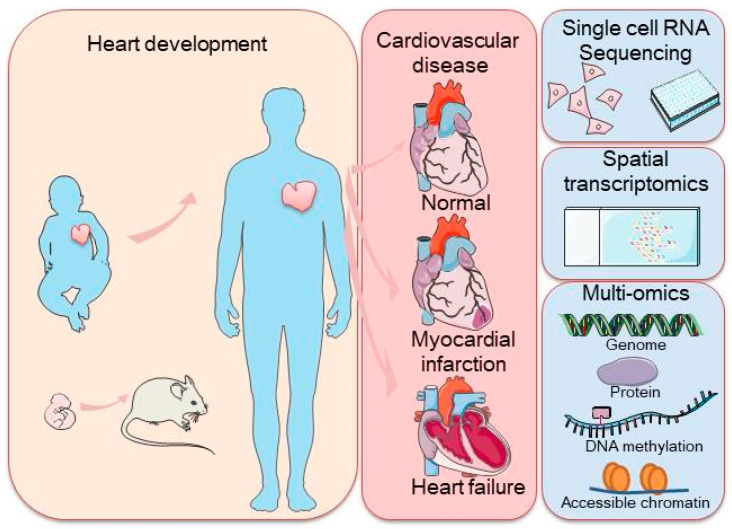
Schematic representation of a commonly used application of single-cell sequencing (scRNA-seq) in the mammalian heart and single-cell transcriptome sequencing methodologies, including scRNA-seq, spatial transcriptome profiling, and multi-omic sequencing. The left panel represents heart development with arrows indicating the transition from embryonic to adult stages, with a focus on heart health. The right panel highlights cardiovascular disease stages, along with cutting-edge technologies such as single-cell RNA sequencing, spatial transcriptomics, and multi-omics approaches used to study the genome, proteins, DNA methylation, and chromatin accessibility.

As this field continues to evolve, the integration of scRNA-seq with other advanced technologies is expected to further accelerate our understanding of cardiac biology and disease, offering new opportunities for the diagnosis and treatment of cardiovascular diseases through precision medicine. In this review, we summarized the insights gained from scRNA-seq studies on the mammalian heart and discuss additional techniques that can complement these findings, offering a deeper understanding of the cellular and molecular mechanisms driving cardiac development, maintenance, and pathology. Finally, we explore future challenges and opportunities for scRNA-seq and related methods in advancing our knowledge of the heart (Figure 1).

## 2. The Application of scRNA-Seq in Understanding Cardiovascular Development and Disease

Single-cell sequencing has gained increasing popularity in the field of heart development and disease over the past several years. Its capability to profile gene expression at single-cell resolution is frequently leveraged to identify rare cell subpopulations and perform cell trajectory analysis, rendering the technique extremely suitable for studying embryonic organ development and disease onset/progression when cell states and fates dynamically change, with the most drastic alterations often occurring within a very limited time window. The application of scRNA-seq has already deepened our understanding of the origins and fates of mammalian heart cells, the regulatory factors and networks orchestrating development and diseases, as well as the diversity of cardiac cells and their interconnections.

### 2.1. Pioneering scRNA-Seq Studies in Heart Development

In 2016, the first reports using scRNA-seq in heart development originated from the laboratories of Dr. Sean Wu [15] and Dr. Christine Seidman [16], pioneering in the spatial and temporal dissection of the cellular composition of the developing mammalian heart. Li et al. [15] generated single-cell transcriptomic profiles from 2233 embryonic mouse cardiac cells (embryonic day (E) 8.5–E10.5) by Anatomical Transcription-based Legend from Analysis of Single-cell RNA-Sequencing (ATLAS-seq). Using a random forest algorithm, they predicted, with >91% accuracy, the anatomical locations of individual cardiomyocytes during development, including lineage-traced cells marked by insulin gene enhancer protein ISL1. In a similar vein, DeLaughter et al. [16] also profiled single cells from embryonic mouse hearts (E9.5–E18.5) as well as postnatal hearts, but focused more on uncovering stage-specific cardiomyocyte transcriptional programs during embryonic and postnatal maturation and identifying lineage-specific gene expression that underlie early cardiac development (Table 1).

### 2.2. Cardiovascular Progenitor Cells (CPCs) and Epicardium-Derived Progenitor Cells (EPDCs) in Heart Development

Much of the subsequent work on embryonic heart development pivoted around cardiovascular progenitor cells (CPCs) that may hold the answer to cardiogenesis. For example, scRNA-seq of wild-type and *Mesp1*-null CPCs demonstrated that *Mesp1* governs exit from pluripotency and cardiovascular lineage specification. The spatial and temporal heterogeneity found within *Mesp1*+ cells are functionally relevant, and they are indicative of the lineage and spatial segregation of progenitor cells. In an effort to delineate molecular regulators of CPCs into specific lineages, de Soysa et al. [17]. identified *Hand2* as a specifier of outflow tract (OFT) cells. Loss of *Hand2* resulted in OFT specification failure, as well as in the inability of the right ventricle to differentiate and migrate properly [22]. Sereti et al. [18] identified proliferative Mesp1- and Nkx2-5-expressing cardiac progenitors as the major source for new cardiomyocytes, whereas α-MHC-expressing cardiomyocytes, although still proliferative at E9.5, almost loose this ability by E12.5. ScRNA-seq helped uncover heterogeneity in the proliferative potentials of α-MHC-expressing cardiomyocytes in the early stages of development. The epicardium is the outermost epithelial lining of the heart that is also a source of multipotent cardiac progenitors [23]. Epicardial cells undergo epithelial-to-mesenchymal transition (EMT) giving rise to epicardium-derived progenitor cells (EPDCs) that migrate into the myocardium and participate in cell-type formation and myocardial vascularization. Single-cell transcriptomic profiling coupled with pseudotime analysis of embryonic Lats1/2 wild-type and knockout hearts revealed that Lats1/2-deficient cells failed to differentiate into cardiac fibroblast, highlighting the crucial role of Lats1/2 in the regulation of these processes [24]. By analyzing Isl1- and Nkx2-5-labeled CPCs at the single-cell level, Xiong et al. discovered that CPCs in the first and second heart fields (FHF and SHF) undergo a distinct transcriptional program to achieve differentiation into cardiomyocytes. They proposed an intraorgan crosstalk model in which early FHF cardiomyocytes instruct the migration of SHF CPCs through chemotaxis that is regulated by Nkx2-5. Epigenetic marks provide an additional guide for cell-subtype identification and shed light on underlying gene regulatory networks in early heart development, which together are essential for a comprehensive understanding of CPC fate determination. Jia et al. comprehensively characterized CPCs marked by Nkx2-5 or Isl1 expression from E7.5 to E9.5 mice using scRNA-seq and transposase-accessible chromatin profiling (ATAC-seq) and discovered that, while Isl1 is absolutely critical for CPC fate bifurcation into endothelial and cardiomyocyte lineages, Nkx2-5 exclusively directs a cardiomyocyte cell fate in CPCs.

Continued efforts in investigating mammalian heart development culminated in the generation of the first single-cell atlas that systematically describes the spatial characteristics and cellular heterogeneity of the human heart at three developmental stages of the first trimester: 4.5–5, 6.5, and 9 weeks after conception [21]. They clarified the spatial distribution and regional heterogeneity of different cell types during the embryonic development of the human heart. Developing cardiomyocytes comprised three populations, namely atrial, ventricular, and *Myoz2*-expressing cardiomyocytes, the latter not being restricted to a specific anatomical region. Major differences with regard to the spatial location and functional specificity were found to exist between single-cell clusters of the epicardium and EPDCs, four fibroblast-like cell populations, and two endothelial cell subtypes. Ultimately, they visualized their results by integrating spatial information to generate a three-dimensional (3D) transcription map, demonstrating the capability of single-cell transcriptomics to reproduce minute details and reconstruct cellular dynamics along with their spatial arrangements that were previously impossible with conventional techniques.

### 2.3. Cardiomyocyte Heterogeneity and Disease-Driven Changes

The earliest single-cell-level sequencing studies of the adult heart started to emerge in 2017 [25,26,27,28], when either non-cardiomyocytes (e.g., immune cells, fibroblast) or cardiomyocyte nuclei were sequenced. Recent advances in single-cell RNA sequencing (scRNA-seq) have allowed for detailed analysis of leukocytes from both infarcted and non-infarcted mouse hearts, uncovering critical cellular responses to myocardial infarction. Specifically, it has been revealed that following ischemic cell death, macrophages engulf cell debris, which in turn activates interferon regulatory factor 3 (IRF3) and triggers the production of type I interferons (IFNs) [26]. This pathway is pivotal in driving the lethal response associated with myocardial infarction, highlighting the significance of immune cell interactions in cardiac injury and repair. ScRNA-seq has provided new insights into the role of cardiac fibroblasts, a crucial cell population known for its transcriptional heterogeneity. These fibroblasts perform various essential functions that vary depending on the heart’s condition. Through the scRNA-seq analysis of 10,519 non-cardiomyocytes from mouse hearts, Skelly et al. have mapped out a complex intercellular communication network among all cardiac cell types. Among these, fibroblasts emerged as the most communicative, underscoring their critical role in maintaining heart function and responding to injury [27]. Schafer et al. [28] found that IL-11 RNA expression was positively correlated with the number of myofibroblasts. The scRNA-seq experiment conducted in a fibrosis-susceptible mouse model identified a subset of IL-11-positive fibroblasts, indicating that IL-11 plays an important role in fibroblast differentiation into myofibroblasts in cardiovascular diseases. In mice and humans, Christina et al. [29] found that scRNA-seq shows that IL-6 is primarily expressed by fibroblasts and is regulated by T cell-derived adenosine. The purinergic metabolic cooperation between fibroblasts and T cells is a mechanism that regulates cardiac IL-6 formation. See et al. performed single-cardiomyocyte nuclear RNA-seq on healthy and failing hearts and uncovered the heterogeneity of the CM transcriptomic stress response, suggesting an opportunity to facilitate cardiac repair by targeting long intergenic non-coding RNAs (lincRNAs). Nicin et al. [30]. performed a snRNA-seq of the cardiac septum from five patients with cardiac hypertrophy. Unsupervised clustering of a total of 88,536 nuclei revealed 19 distinct clusters. They highlight the importance of intercellular crosstalk in disease pathogenesis. Reduced cell crosstalk in the hypertrophied heart may be because Eph receptor tyrosine kinases, particularly EPHB1, were significantly downregulated in cardiomyocytes. EPHB1 activation by ephrin (EFN) B2 was reduced, mainly expressed by endothelial cells. EFNB2 silences induced hypertrophy.

With hand-picking viable cardiomyocytes, fluorescence-activated cell sorting (FACS)-based techniques, or nanoplate-based systems, adult cardiomyocytes were sequenced [31,32], which provided insights into cardiomyocyte heterogeneity in the adult heart, as well as cardiac abnormalities, specifically ischemia and hypertrophy. Recent studies have highlighted a significant limitation when applying SnRNA-seq to cardiomyocytes, particularly due to the presence of multinucleated cells. In adult cardiomyocytes, the existence of multinucleated cells can distort cell clustering and analysis when using snRNA-seq, as this technique relies on single nuclei, which may not accurately represent the properties of the entire cell population. However, this issue has been addressed through the sequencing of intact single cardiomyocytes, which reveals that despite their mono- or multinucleated status, these cells tend to express similar sets of genes. This finding is crucial as it alleviates concerns regarding the distortion of gene expression analysis in cardiomyocytes, ensuring more reliable results in studies of cardiac biology.

Although adult cardiomyocytes are already heterogeneous [1,31], an additional level of heterogeneity can be induced by disease conditions, such as by hypoxia in the hypertrophic heart [33]. By use of Smart-seq2-based scRNA-seq on a mouse model of pressure overload-induced cardiac hypertrophy, Nomura and colleagues [32] elucidated heterogeneous gene expression as well as the trajectories of hypertrophying cardiomyocyte, unveiling a central role of p53 in promoting conserved pathogenic gene programs in cardiomyocytes. The same model was also used to delineate the spatiotemporal interplay of different cardiac cell types during the progression of cardiac hypertrophy [34]. Macrophage subtype switching at middle-stage adaptive hypertrophy determined cardiac outcome, whose pharmacological inhibition was sufficient to ameliorate the deleterious consequences related to hypertrophy. Gladka et al. applied scRNA-seq to dissect cell-type-specific alterations during ischemic injury, and they identified cytoskeleton-associated protein 4 (CKAP4) as a novel marker for activated fibroblasts, which exhibited clinical relevance. As a key player in the inflammatory response, macrophages are also known to mediate major responses to ischemic injuries [35,36,37,38]. Dick et al. [39] studied the heterogeneity of cardiac macrophages under steady state and their plasticity after infarction, and they showed that resident macrophages in the infarct area were significantly reduced after infarction. This type of macrophage exhibited a spatially localized cardioprotective effect through slow in situ proliferation. In hypertrophic cardiomyopathy, Wehrens et al. [40] identified six subpopulations of cardiomyocytes, one of which exhibited a greater abundance expression of natriuretic peptide A (NPPA). Twenty-two regulons were consistently identified, implying that NFE2L1 and MAFK are cooperatively induced in a set of CM to drive a gene program involved in sarcomere organization and muscle contraction (Table 2).

### 2.4. Charting the Cellular Landscape of the Adult Human Heart

Charting the cellular landscape of the adult human heart is a daunting task, not only because of limited tissue availability and the complex anatomy of the heart, but also owing to the large size and fragility of adult cardiomyocytes. Wang et al. created the first cellular map of the adult human heart (http://sklcvd.fuwaihospital.org/wanglab/data (accessed on 3 July 2024)), where they analyzed and characterized a total of 11,492 single cells from different cardiac regions and under various health conditions (i.e., normal, heart failure of distinct etiologies, recovery from heart failure). They discovered that the cardiomyocyte microenvironment, particularly endothelial cells, has a profound impact on cardiomyocyte function. Injecting ACKR1^+^ endothelial cells into infarcted hearts can mitigate the decline in cardiac function [1]. Subsequently, two other large-scale sequencing studies of the adult human heart were published, both of which leveraged single-nucleus sequencing for cardiomyocytes. Tucker and co-workers [2] sequenced 287,269 nuclei from the four chambers of the normal human heart, and they identified nine major cell types as well as over twenty cellular subtypes. Intersection of their scRNA-seq data with genome-wide association studies revealed the most relevant cell types for cardiovascular disease. For example, CMs were specifically enriched in atrial fibrillation, as well as PR and QT interval abnormalities, whereas adipocytes seemed to play a major role in low-density lipoprotein cholesterol disorders. On the other hand, integrating scRNA-seq data with the druggable genome hinted at adipocytes, CMs, and fibroblasts as possibly being the most tractable cell types that could be exploited for therapeutic targeting. Litviňuková et al. [11] presented comprehensive transcriptomic data on six distinct cardiac regions, using both scRNA-seq (for non-cardiomyocytes) and snRNA-seq (for cardiomyocytes). Beyond a detailed description of the cellular heterogeneity, they identified chamber and sex-specific differences in the cellular composition of the heart. They revealed atrial and ventricular cellular types with different developmental origins and characteristics, and they showed the complexity of macrophages and inferred the paracrine circuit that maintains cardiac homeostasis. Multiplex single-molecule fluorescence in situ hybridization (smFISH) was used to highlight the spatial arrangements and relationships of select cell populations. This huge reference framework expands our knowledge of the human heart and could be utilized, in conjunction with the cell map of the developing heart [21], to extract clues for cardiac maturation and regeneration.

### 2.5. Postnatal Heart Development and Maturation

Compared to the large body of single-cell work dedicated to deciphering cardiogenesis, embryonic cardiac development, adult cardiac homeostasis, and dysfunction, few studies were directed at postnatal development and maturation [43]. Recent studies utilizing massively parallel single-nucleus RNA sequencing (snRNA-seq) on postnatal day 6 (P6) and day 10 (P10) mouse hearts have provided significant insights into the development of cardiomyocytes during early postnatal stages [42]. The use of sNucDrop-seq technology has revealed a notable decrease in the proportion of proliferating cardiomyocytes alongside a simultaneous increase in the percentage of mature cardiomyocytes. This shift indicates that cardiomyocytes undergo active differentiation and maturation within this critical developmental window. Despite these findings, the specific factors driving cardiomyocyte maturation remain only partially understood. Understanding these maturation factors is crucial for advancing our knowledge of heart development and could have significant implications for regenerative medicine and the treatment of heart diseases. Wang et al. [41] employed scRNA-seq and unraveled cardiac fibroblast subtype switching to be a critical determinant of postnatal cardiomyocyte maturation in vivo, underscoring the involvement of cellular crosstalk in postnatal heart maturation.

### 2.6. Limitations and Future Directions for Single-Cell Techniques

The widespread application of single-cell and single-nucleus transcriptomic profiling has provided researchers with access to an unprecedented resolution of cellular gene expression. However, the transcriptome is a relatively downstream readout in the regulatory network that defines cell fate and function. For elucidation of regulatory mechanisms, one may wish to delineate chromatin states. On the other hand, when understanding cellular function, one might hope to correlate mRNA expression with protein expression. In addition, with scRNA-seq only, cellular maps are created from physical dissection of tissue at best, such as breaking down the heart into four cardiac chambers. Alternatively, orthogonal molecular validations of selected areas or cell types are possible, but they are extremely low-throughput. Therefore, these maps are likely too rudimentary to gain sufficient spatial information on the cellular arrangement and environmental components. Hence, it was these unmet needs that brought forth the combinatorial analyses of single-cell transcriptomes and other molecular entities and indices, including genomes, methylomes, chromatin opening states, proteomes, and spatial distribution of individual cells (spatial transcriptomics).

## 3. Combination of scRNA-Seq with Other Single-Cell-Level Assessments

As illustrated above, scRNA-seq is commonly used to elucidate the cellular composition of an organ. However, despite its contributions, scRNA-seq faces limitations in capturing the spatial localization of cells within the heart tissue and the dynamic intercellular interactions crucial for comprehensive analysis. This gap in spatial and intercellular information has left several aspects of molecular changes, particularly their relationships and mechanisms, still unresolved. As the heart is the first fully functional organ during embryonic development, understanding these complex processes at a molecular level is essential for advancing our knowledge of heart physiology and disease. The differentiation of the cardiac tube from the mesoderm, the formation of four heart cavities through bending, and the septation of the outflow tract into the trunks of the aorta and pulmonary artery are processes that have been well documented at a general level, yet many molecular intricacies remain to be explored.

### 3.1. scRNA-Seq and Spatial Transcriptomics

**Figure 2:**
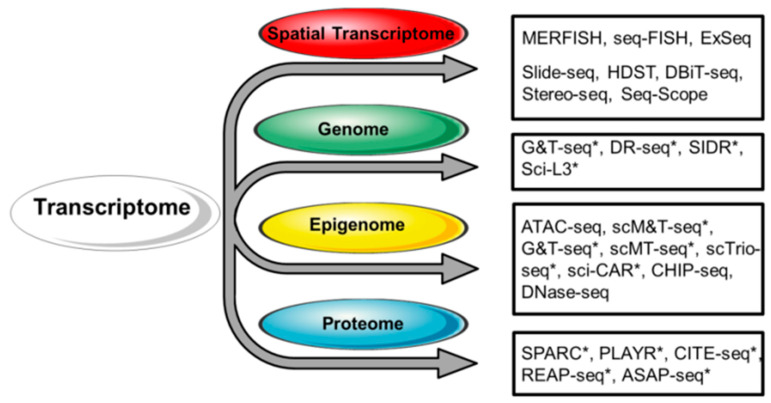
Strategies for the integrated analysis of single-cell transcriptome and other single-cell omic approaches. Respective techniques are shown on the right. Single-cell multi-omic approaches are indicated with an asterisk (*). At present, scRNA-seq can be successfully combined with the analysis of the spatial distribution of RNA, the genome, chromatin accessibility, DNA methylation, and targeted protein expression.

Most solid tissues are composed of different cell subgroups, with their spatial location and function deeply intertwined. Cell communications via juxtacrine and paracrine mechanisms are essential for the function of normal tissues, while their distortion (e.g., abnormal spatial positioning of cells) can lead to diseases. Previous work has repeatedly demonstrated the important role of cell-to-cell communication and interactions in heart development and diseases [1,34,41]. In traditional scRNA-seq, predicted interactions are usually orthogonally validated by a set of biochemical validations, such as immunofluorescence and immunohistochemistry, which can be limited in throughput and resolution. However, to depict a cellular map and to decipher cellular communities that carry out biological functions, one would need to further increase dimensionality and spatial resolution. For this reason, the integration of spatial transcriptomics (ST) (Table 3) and scRNA-seq has become a rapidly developing direction (Figure 2).

The spatial transcriptome shows the spatial distribution of the single-cell transcriptome landscape in the entire organization by retaining the original location information. At present, there are two major methods for determining the original position and molecular expression of a single cell. The first is the method of positioning hundreds of genes in intact tissues by high-density RNA imaging (HPRI), including in situ sequencing (ISS) [44] and in situ hybridization [45] (e.g., MERFISH (multiplexing error-robust fluorescence) and seqFISH (sequential fluorescence)). BaristaSeq [46] and STARmap [47], both derived from ISS, improve sensitivity and throughput by increasing base read length and reducing the reverse transcription steps, respectively. MERFISH technology combines labeling, continuous imaging, and other technologies to improve detection throughput, and it uses binary barcodes to replace single-molecule labels to improve sensitivity [48]. In contrast, seqFISH, which uses successive rounds of hybridization and imaging, is expensive and time-consuming, but it can achieve high-resolution imaging of RNA [49]. Recently, a combination of fluorescence in situ sequencing technology (FISSEQ) [50] and expansion microscopy (ExM), termed expansion sequencing (ExSeq), was used for cell-type mapping in the mouse brain and human tumor tissue. Two versions of ExSeq, untargeted and targeted, were developed, which reached a resolution of nanoscale subcellular compartmentalization at the system level [51].

The second class of method is based on next-generation sequencing (NGS), where the position information is encoded on the transcript before NGS [52]. This technology has evolved from the original 100 μm diameter of each spot on a slide to an updated version of a 55 μm spot diameter and more than 10,000 transcripts per spot, demonstrating higher resolution and sensitivity [53]. Alternatively, randomly barcoded beads were deposited onto rubber-coated glass coverslips (Slide-seq [54], 10 μm resolution) or into a hexagonal array of 2 μm wells (high-definition spatial transcriptomics (HDST) [55]), to even further enhance resolution. Recently, more advanced methods, including DBiT-seq [56], Stereo-seq [57], Seq-scope [58], and PIXEL-seq [59], keep pushing towards the limits of resolution.

Despite these staggering technological advancements, few studies have incorporated them into the revelation of heart biology and disease. Combining three different technologies, i.e., NGS-based ST, scRNA-seq, and ISS, Asp et al. [21] characterized the spatiotemporal expression patterns of genes during human heart development and the spatial organization of cell types in human embryonic hearts, and they created a cellular atlas of human cardiac development, with which they established an open network resource. However, the study has several limitations, including failure to detect rare cell types and identify spatiotemporal cell–cell interactions due to the limited number of cells detected (3717 cells), limited number of genes being used in the ISS panel, and low resolution of the ST technique (3115 spots containing almost 30 cells per spot). Mantri et al. [60] analyzed four key stages of ventricular development in the chicken heart. Single-cell transcriptome data of 22,315 cells and spatial transcriptome data covering more than 6800 spots were generated. By capturing more cell phenotypic heterogeneity and showing several patterns of epicardial and vascular development, they created a spatiotemporal cell diagram of chicken heart development. Their findings show that the combined use of scRNA-seq and ST is suited to study the interaction between cell differentiation and morphogenesis and achieve high-resolution readout of spatial gene expression heterogeneity and even spatial cell heterogeneity.

Spatial transcriptome data can be used to reveal cell-to-cell communications and biological functions in different tissues. In an Alzheimer’s disease (AD) mouse model, Chen et al. [61] used ST to reveal the transcriptional changes that occurred in the 100-micron-diameter tissue domain around amyloid plaques, and they verified them by orthogonal ISS methods. They identified the plaque-inducing gene network and the oligodendrocyte gene response in AD, and they generated a large data set of transcriptional changes in mouse and human brains with increased amyloid pathology. To date, ST has not been effectively employed to study cardiac disorders, despite mounting evidence that cellular crosstalk plays key roles in the heart. This technique might be particularly useful for structural cardiac defects, such as the pathogenesis of Tetralogy of Fallot, or processes that involve the spatial rearrangement of cells, such as endocardial precursor migration. From a clinical standpoint, ST could potentially compliment current pathological examinations to aid diagnosis. Analysis can be focused on characterizing the few regions of interest that drive disease-related phenotypes. Understanding the spatiotemporal gene expression patterns in the cell types that influence the disease may support prognosis, and it is of great significance to precision medicine.

### 3.2. scRNA-Seq and Epigenomics

The chemical modification of DNA and histones and the physical conformation of DNA in the nucleus define the epigenome. Because genomic DNA is tightly wrapped around histones, their covalent modifications are needed to make DNA regulatory elements and promoters available for transcription, thereby defining the transcriptome. Therefore, many biological processes are closely related to the epigenetic regulation of chromatin accessibility. In the heart, epigenetics plays a key role in the cell specialization of the sinoatrial node during development [62], targeted therapy of cardiomyopathy [63], and cell differentiation and transcriptome remodeling during cardiac hypertrophy [64]. During early embryonic development, the specialization of cardiac pacemaker cells requires a combination of cell-type-specific transcription regulators to activate the expression of key effector genes by combining with DNA regulatory elements including enhancers and promoters [65]. Similarly, during the pathogenesis of heart failure, the transcriptomes of cardiac fibroblasts and muscle cells undergo programmed changes that occur via histone acetylation and methylation of genes related to heart development and diseases. Cardiomyopathies can be either hereditary (or familial) or non-hereditary (or secondary). So far, the major focus of studies regarding the pathogenesis of cardiomyopathies has been on genetic factors. However, accumulating evidence shows that genetic mutations are not always related to the diagnosis of CMP, suggesting that other mechanisms, such as epigenetics, may play a role in the occurrence or progression of cardiomyopathies [66] (Table 4).

Single-cell epigenome profiling is capable of measuring DNA methylation, chromatin accessibility, and chromatin conformation. Chemical and physical modifications of DNA are established during development and regulate gene expression throughout life. These markers influence gene expression by directing the binding of transcription factors to specific genomic regions, which can enhance or reduce the transcription of nearby or remote genes. As a supplement to the single-cell transcriptome, it provides in-depth understanding of cell-type-specific gene expression regulation. Epigenomic technologies such as assay for transposase-accessible chromatin (ATAC-seq) [66], ChIP-seq [67], Hi-C [68], and DNase-seq [69] have recently been developed to assess chromatin structure at single-cell resolution. Although the sensitivity of traditional ChIP-seq is low, recent developments such as nano-ChIP [70], iChIP [71], MOWChIP [72], and SurfaceChIP [73] have greatly reduced the amount of cells. Single-cell ChIP using droplet separation (Drop-ChIP) [74] analyzes post-translational histone modifications that can be captured at single-cell resolution and have a coverage of 1000 unique reads per cell.

Joint analyses by scATAC-seq (assay for transposase-accessible chromatin using sequencing) and scRNA-seq facilitate the characterization of correlations between highly accessible chromatin regions and the expression of corresponding transcripts and thus provide insights into the gene regulatory mechanisms of specific cell subpopulations. Their combination and mutual validation allows for analysis of the layered regulatory networks from DNA to RNA at a specific time and space, and, when coupled with phenotypic and functional analysis, is able to delineate the mechanisms of the genomic regulation of a particular phenotype. ScATAC-seq has been applied to characterize chromatin accessibility and putative regulatory elements driving cardiogenesis. As mentioned above, Jia et al. characterized mouse cardiac Nkx2-5 and Isl1 + CPCs using scRNA-seq and scATAC-seq, providing maps of transcriptional and epigenetic regulation during CPC fate decisions. In an in vitro model of direct cardiac reprogramming from cardiac fibroblasts, scATAC-seq helped unveil changes in the epigenetic landscapes during the early phases of transdifferentiation [75]. Integration of ATAC-seq data with scRNA-seq data yielded active transcription factors, including Smad3 and Fos, that were critical to reprogramming efficiency. The biological function of chromatin remodelers are particularly well suited to be analyzed by ATAC-seq. Liu et al. [76] combined whole-genome-wide ATAC-seq, ChIP-seq with scRNA-seq to reveal the role of ARID1A, a subunit of the SWI/SNF chromatin remodeling complex, in early human cardiac and neural development using a human embryonic stem cell (hESC) model. Instead of using scATAC-seq, the authors employed bulk ATAC-seq in differentiating WT and ARID1A knockout hESCs, as well as ChIP-seq in WT hESCs, to assess chromatin accessibility and ARID1A occupancy on cardiogenic versus neurogenic genes, respectively. It is the joint application of these methods that illustrated the mechanisms by which ARID1A coordinates early human cardiogenesis and neurogenesis. Xiang et al. [77]. conducted an integrated analysis of scRNA-seq, scATAC-seq, bulk ATAC-seq, and miRNA-seq data on the hearts of mice with heart failure (HF). The scRNA-seq analysis identified five major cell types, while the combined analysis of ATAC-seq and miRNA-seq data revealed the transcriptional activation of genes involved in immune responses and disrupted miRNA expression in immune cells. The scATAC-seq analysis further uncovered the upregulation of genes related to nitric oxide (NO) biosynthesis in endothelial cells during heart failure.

From a translational perspective, the concomitant use of scATAC-seq and scRNA-seq may identify potential therapeutic targets. Dr. Eric Olson’s laboratory [78] created a database of injury-responsive gene regulatory networks that determine heart regeneration and cardiac remodeling, by scRNA-seq and scATAC-seq profiling of 17,320 and 30,520 single cells, respectively, from regenerative and non-regenerative mouse hearts. To gain functional insight, they overlapped cell-type-specific open chromatin regions with previously characterized cardiovascular enhancers. They uncovered a distinctive response of epicardial cells during neonatal heart regeneration that may suggest potential therapeutic targets for preventing cardiac fibrosis upon injury. In addition, concurrently studying the epigenome and transcriptome of the heart at single-cell resolution may also help us discover clinically relevant biomarkers of heart diseases. The usefulness of cardiac-specific plasma protein biomarkers such as b-type natriuretic peptide (BNP), N-terminal pro-b-type natriuretic peptide (NT-proBNP), and cystinosin (cTns) in the diagnosis of cardiomyopathy is questioned because they may not be sufficient for early detection or better risk stratification of genetic diseases [79], whereas the use of other alternatives, such as circulating miRNAs, has been increasing in the past few years [80]. Although not yet implemented for cardiovascular diseases, studies in other disease types may shed light on their usefulness in biomarker discovery [81,82].

### 3.3. scRNA-Seq and Proteomics

From ion channels and gap junctions that mediate electrical conduction, to myosin chains and actin filaments that perform physical contraction, proteins are the ultimate executors of cardiomyocyte function. Although the transcriptome has long been used as an approximation of the proteome due to ease of analysis, one still needs to be aware of their fundamental differences. Protein abundance and turnover, conformational changes, and their post-translational modifications are not reflected in the transcriptome. Therefore, proteomic analysis of a large number of cell types is expected to give us a macroscopic view of how proteins work when the heart performs these special functions. However, owing to the technical challenges associated with single-cell proteomics, multiplexed analysis of single-cell transcriptomics and proteomics per se has never been successfully implemented. Either the bulk proteome or single-cell-level expression of a panel of proteins was inquired into in lieu. Attempts at the latter will be discussed in the next section.

Several joint scRNA-seq and protein studies were conducted in non-cardiac systems, including the liver [83], skin [84], and ileum [85]. In the cardiovascular arena, Winkels et al. [86] leveraged scRNA-seq to elucidate the composition of leucocytes in healthy and atherosclerotic mouse aortas, and they revealed unexpected heterogeneity of leucocytes in atherosclerosis, the phenotypes of which were validated by flow cytometry (CyTOF). The clinical relevance of aortic leukocyte populations was assessed by CyTOF with an anti-human antibody panel. This integrated approach helped improve our understanding of leucocyte diversity and dynamics in atherosclerosis. Ma et al. [87] explored the markers and molecular mechanisms of left heart dysplasia (HLH) through scRNA-seq and quantitative proteomics. Three central genes (*MMP2*, *B2M*, and *COL5A1*) were identified, which exhibited high expression in the left (LV) and right (RV) ventricles of HLH patients compared with the control group. Shen et al. [88]. used scRNA-seq and mass cytometry to examine monocyte-derived macrophages in mice following myocardial ischemia reperfusion (MIR). They identified a distinct S100a9^hi^ macrophage subtype that infiltrates the heart as early as 2 h after reperfusion and activates the Myd88/NFκB/NLRP3 signaling pathway, thereby amplifying the inflammatory response. Targeting S100a9 can effectively reduce acute inflammatory injury and prevent the progression of fibrosis, including myocardial matrix remodeling (MMT).

The coupling of RNA of protein measurements at the single-cell level not only promotes basic research but may also be a useful way to develop biomarkers for disease diagnosis and monitoring. As we are continually refining disease classification, severity, and phenotype based on data from single-cell studies, biomarkers derived from these findings are expected to be more specific, sensitive, accurate, and may even distinguish between individuals. It is worth noting that post-translational modifications (PTMs) endow proteins with immense functional diversity, and they are very often disease-relevant (e.g., kinase phosphorylation cascades and histone modifications). Thus, the incorporation of PTM-specific proteomics could prove highly informative.

## 4. Multi-Omic Study of Single Cells

As discussed above, to achieve simultaneous measurements of various parameters, a sample, such as a single-cell suspension, can be divided up and allocated for different purposes. However, the integration of data from different pools of cells, although quite informative, is far from being ‘definite’. For example, one can relate RNA abundance to chromatin accessibility through bioinformatic interpolation, but the data are not acquired from the same single cell. To establish a conclusive relationship between two or more modalities, measurements need to originate from the same cell. Therefore, the field is actively moving towards the use of single-cell multi-omics (scMulti-omics) to seek a deeper understanding of the coordinated interplay of multiple types of molecules (such as DNA, RNA, and protein) at the single-cell level, and to understand the states and functions of cells through multi-dimensional analysis.

Compared with single-omic data based on multi-channel molecular readings, the core components of single-cell multi-omic analyses are single-cell separation, barcoding, and sequencing technology to measure multiple types of molecules from the same cell. Prior to obtaining molecules of interest, cells are randomly collected from a heterogeneous population. The success of the collection step is critical to preserving accurate representations of DNA, RNA, and proteins within the cell for downstream measurements. Then, single cells are processed according to the desired protocol to separate multiple types of molecules from the same cell. The molecules of interest can be targeted and captured either with or without physical fractionation or splitting of the cell. At present, the workflow of single-cell genome and transcriptome analysis is relatively well established, while other types of scMulti-omics are still under continuous development and optimization. Due to the lack of general single-cell multi-omic applications in cardiovascular research, we will mainly focus on brief introductions of the different methodologies below.

### 4.1. Single-Cell Genome and Transcriptome

One way to concurrently examine genomic DNA (gDNA) and mRNA is to obtain them from the nuclear and cytoplasmic fractions of a single cell, respectively. Lin et al. [89] reported an integrated microfluidic chip platform that captures and lyses single cells, respectively, extract nuclear gDNA and cytoplasmic mRNA, and they performed reverse transcription of mRNA, followed by whole-pool amplification of both complementary DNA (cDNA) and gDNA. Although the original report only showed data for the entire transcriptome, but not the entire genome, the system enabled real-time imaging, scaled operation, and low reagent consumption. In a similar fashion, Han et al. [90] reported another method for the simultaneous isolation of gDNA and RNA (SIDR) from single cells. This method uses hypotonic lysis to disrupt the plasma membrane to allow for diffusion of RNA, but it maintains the integrity of the nuclear layer. DNA and mRNA may also be extracted without physical fractionation of the cell. MacAulay et al. [91] devised a method, named G&T-seq, for separating and sequencing genomic DNA and full-length mRNA from single cells. In this protocol, polyadenylated (poly (A)) mRNA is captured by magnetic beads coated with biotinylated oligo-dT primers, and it is reverse-transcribed using a modified Smart-seq2 protocol. The mRNA-depleted gDNA-containing lysate is then harvested, concentrated, purified, and subjected to whole-genome amplification (e.g., PicoPLEX, MDA and MALBAC). Contemporarily, Dey et al. [92]. introduced a method called gDNA-mRNA sequencing (DR-Seq) that also circumvents the physical separation of a cell’s contents. Here, mRNA is first reverse-transcribed into single-stranded cDNA. Following seven rounds of quasilinear whole-genome amplification of both gDNA and single-stranded cDNA, the sample is split in half, and each set of the molecular entity is amplified separately. More recently, sci-L3, a single-cell sequencing method that combines single-cell combinatorial indexing (‘sci-’) and linear (‘L’) amplification, was developed, which can be generalized to perform simultaneous profiling of the genome and transcriptome (sci-L3-RNA/DNA). In this protocol, formaldehyde-fixed nuclei are distributed into the wells of 96-well plates, and a first round of DNA barcoding (by Tn5 transposon insertion) and RNA barcoding by in situ cDNA synthesis with a barcode and UMI-carrying polyT primer is performed to distinguish the nucleic acids [93]. Despite its relatively low genome coverage, the authors envision this sci-L3 scheme to be adapted to ATAC-seq, bisulfite-seq, and Hi-C in the future (Table 5).

Because these methods can directly determine the correlation between copy number variations and gene expression, they may be applied to delineating the transcriptional consequences of chromosomal aneuploidies and interchromosomal fusions, improve the sensitivity of cell-subtype identification and lineage tracing, and even potentially characterize coding single-nucleotide variants at single-cell resolution. Gong et al. [94]. utilized genetic lineage tracing in mice and scRNA-seq analysis to study the cells and intercellular communication in allogeneic transplanted hearts. They found that cardiac lymphangiogenesis primarily originates from recipient cells, and that activated fibroblasts promote post-transplant lymphangiogenesis through VEGFD/VEGFR3, MDK/NCL, and SEMA3C/NRP2 pathways, contributing to graft survival.

### 4.2. Single-Cell Epigenome and Transcriptome

The first reported method for combining DNA methylome and transcriptome analysis is scM&T-seq [95], in which the protocol for mRNA capture, amplification, and sequencing is the same as that in G&T-seq. At the same time, the gDNA is subjected to single-cell reduced representation bisulfite sequencing (scRRBS) [96]. By contrast, scMT-seq [97] selectively lyses the cell membrane to release RNA and physically separates the intact cell nucleus from the cell lysate. Single-cell nuclei are collected with a micropipette for scRRBS, while mRNA in the lysate is amplified via a modified Smart-seq2 protocol. Hou et al. [98]. reported single-cell triple-omic sequencing (ScTrioseq) to simultaneously analyze the DNA methylome, genome, and transcriptome in a single cell, in which the mRNA-containing supernatant and the DNA-containing precipitate are analyzed by scRNA-seq and scRRBS, respectively. Likewise, this trio has been applied to primary diffuse gliomas, markedly enhancing the resolution of single-cell identification of copy number alterations [99]

In addition, methods to interrogate other aspects of the cell’s epigenome have emerged, including scATAC-seq, scDNase-seq [100], and scChIP-seq [67], further expanding the toolkit for the concurrent assessment of the epigenome and the transcriptome. Cao et al. [101] developed sci-CAR for jointly profiling chromatin accessibility and mRNA in single cells, which greatly improved assay throughput compared to preceding methods. In the heart, Wang et al. [102] used scRNA-seq and scATAC-seq to describe the transcriptomic and epigenomic characteristics of cardiac noncardiomyocytes in adult mouse hearts. They uncovered three functionally distinct cardiac fibroblast subpopulations, which are related to cell response, cytoskeleton organization, and immune response, respectively, underscoring the value of combined sequencing in increasing the sensitivity of cell-subtype identification (Table 6).

### 4.3. Single-Cell Protein Expression and Transcriptome

To date, the most widely used single-cell protein detection methods rely on the use of labeled antibodies to target specific proteins. Protein detection by fluorescence microscopy or fluorescence-activated cell sorting (FACS) enables the detection of proteins in individual cells at low throughput (about 10–15 proteins in total). Emerging methods to improve the detection breadth include the use of metal-labeled antibodies, oligonucleotide-labeled antibodies, qPCR, and single-cell mass spectrometry [103], but they are still restricted to hundreds of proteins per cell, and they are therefore not representative of the proteome.

Multiplexing transcriptomics and protein analysis provides the exciting possibility of measuring the abundance and dynamics of RNA and protein in single cells. The most direct method is to use single-cell index FACS to correlate the immunofluorescence signal of a single protein or a small number of proteins with their corresponding transcript levels in the same cell [104]. More advanced methods use FACS and imaging-based methods to simultaneously measure mRNA and protein [105,106,107]. Proximity extension analysis (PEA) in parallel to scRNA-seq employs two antibodies labeled with complementary oligonucleotides to recognize different epitopes of the same protein, which can be amplified and detected by qPCR. An alternative method is to divide the cell lysate into two portions, one for transcript detection and the other for PEA [108]. Using this method, the correlation between transcripts and protein abundances from 22 genes in a single cell can be investigated. A recent study proposed SPARC [109], a method that combines single-cell RNA sequencing with PEA, which can simultaneously measure global mRNA and 89 intracellular proteins in a single cell. The proximity ligation assay for RNA (PLAYR) uses a similar approach to bring two antibody-conjugated oligonucleotides in close proximity to the same protein target. It enables the quantification of multiple specific transcripts in thousands of single cells per second by mass spectrometry-based CyTOF [110]. The most recent technological advancement is the development of new methods, such as CITE-seq [111] and REAP-seq [112], which incorporate whole-transcriptome scRNA-seq and the use of oligonucleotide-conjugated antibodies for single-cell analysis. These methods yielded higher clustering resolution and key insights into cellular function. Mimitou et al. [113] developed ATAC with select antigen profiling by sequencing (ASAP-seq) that pairs chromatin accessibility with surface and intracellular protein expression. They further combined ASAP-seq and CITE-seq to reveal the different regulatory layers of chromatin accessibility, mRNA levels, and proteins. Trzupet et al. [114] quantified the expression of 397 genes at the mRNA level and up to 68 genes at the protein level (via AbSeq technology), shedding light on the biology and dynamics of CD4^+^ T cells of human immune cells. In terms of the throughput of detected proteins, the most comprehensive study profiling proteins and transcripts to date is a total of 12,003 proteins targeted by 13,993 antibodies, by an imaging proteogenomic approach, which together defined the proteome of 13 major organelles [115](Table 7).

In the heart field, Chelko et al. [116] applied CITE-seq to profile gene expression and surface protein markers in Dsg2 mut/mut mice, revealing pro-inflammatory changes in cardiac myocytes, fibroblasts, and CCR2^+^ immune cells. CITE-seq showed that CCR2^+^ cells were recruited to the heart, driving myocardial injury and arrhythmias, and influencing gene expression in myocytes and fibroblasts, highlighting the complex immune–cardiac cell interactions in the pathogenesis of arrhythmogenic cardiomyopathy. Vyas et al. [117] used CITE-seq and single-cell TCR sequencing to identify two transcriptionally distinct CD8^+^ tissue-resident memory T (TRM) cell subsets in epicardial adipose tissue (EAT) that are modulated in atrial fibrillation (AF). Spatial transcriptomics revealed intense inflammatory and fibrotic activity at the EAT–atrial tissue border. The study demonstrated that TRM cells can alter calcium flux in atrial cardiomyocytes and activate inflammatory and apoptotic pathways, indicating that EAT serves as a reservoir of TRM cells that modulate susceptibility to AF. Vafadarnejad et al. [118]. employed CITE-seq and single-cell transcriptomics to characterize the temporal diversity of neutrophils in the blood and heart after murine myocardial infarction. Neutrophils were classified into six distinct clusters with time-dependent changes. Early-stage neutrophils resembled bone marrow neutrophils, while later stages revealed two main subsets, SiglecFhi and SiglecFlow, with unique gene signatures. The SiglecFhi neutrophils were locally acquired in the heart and absent from other tissues. Reducing blood neutrophil influx increased cardiac SiglecFhi neutrophils, suggesting local aging. These findings highlight the dynamic and locally specialized role of neutrophils in heart inflammation. The ever-expanding panels of proteins that are analyzed in conjunction with mRNA at the single-cell level will keep broadening and deepening our knowledge of the heterogeneity, crosstalk, and spatiotemporal dynamics of cells.

## 5. Perspectives and Significance

In 2009, Tang et al. [119] reported the first single-cell RNA sequencing study, opening the doors to an entire realm of biological explorations at single-cell resolution. In recent years, research on improving the depth of single-cell transcriptome sequencing, breadth of genome coverage, and throughput by liquid-handling robots or automated work stations, as well as the versatility of bioinformatic algorithms, has advanced by leaps and bounds. As one of the most dynamically changing and technically most easily profiled molecules among members of the central dogma, mRNA has gained such popularity that it is often seen as a surrogate indicator for biological processes. However, sophisticated as it is, single-cell transcriptome data alone are not sufficient to inform us of upstream regulatory events or downstream phenotypic presentations. Nor do they readily correlate with changes in the metabolome, the latter frequently distorted in pathological conditions. Moreover, in solid organs with intricate structures, represented by the heart, it is highly desirable to spatially locate the transcriptomes of cells to gain insight into their functional specifications and intercellular connections. Consequently, the comprehensive examination of single-cell transcriptomic data alongside various omic disciplines, especially through the methodology of scMulti-omics, constitutes a vigorously evolving domain of inquiry, anticipated to significantly enhance our comprehension of biological systems.

Despite its encouraging future, the application of scMulti-omics is still in its early stages. Many technical and computational limitations need to be overcome in order to improve the content and quality of the information obtained from scMulti-omic analysis. For example, bisulfite treatment can cause DNA damage, affecting the accuracy of readout. During cell processing, such as nucleo-cytoplasmic fractionation, target materials may be lost at different rates, introducing variable bias in subsequent measurements. Furthermore, the number of proteins that can be detected in a single cell remains a technical hurdle, while untargeted single-cell proteomics is not yet established. These and other factors greatly limit the current application of scMulti-omics in cardiovascular research. In addition to these technical difficulties, the computational challenges associated with the integration of multi-omic data are greater than ever. Although single-cell multimodal evaluation alleviates the difficulties of mapping unmatched data, other problems are associated with matched data. Identifying the source for variation can be challenging, because the different modalities already come from the same cell. Feature correspondence is not obvious, since under most circumstances different modalities do not correspond one to one. Additionally, effective visualization for single-cell multi-omic data is immature. Although several algorithm packages exist for single-cell multimodal analysis, they each come with their own limitations that users need to be aware of. For example, naïve approaches (e.g., BREM-SC [120]), which transform the data such that all features end up statistically homogeneous, ignore the biological nature of different modalities. Latent space approaches, such as MOFA and MOFA+, assume that the observed data can be approximated by a weighted linear function of an underlying common latent space, which might not suit non-linear feature relationships. Therefore, there will an increasing need to develop more specialized tools to decode the biological meanings behind such high-dimensional data.

Compared with genomics and epigenomics, single-cell proteomics is still in its infancy, but researchers are making efforts to improve the sensitivity and throughput of single-cell protein detection via mass spectrometry-based approaches. Brunner et al. [121]. developed a workflow that combines miniaturized sample preparation, extremely low flow rate chromatography, and a new trapped ion mobility mass spectrometer. They measured a series of diluted HeLa cell lysates on a quadrupole time-of-flight instrument (TIMS-qTOF), ranging from 25 ng to several single cells. Zhu et al. [122] used a highly sensitive single-cell proteomic method, which can recognize 50–75 proteins in each cell. Emerging technologies, such as single-molecule protein sequencing [123] and single-cell spatial proteomics [124], may further augment the power of protein detection techniques, offering new ways to create high-resolution protein maps of organ systems.

Since 2012, scRNA-seq and related technologies have generated 458 human cell atlases comprising 62.1 million cells, offering unprecedented resolution of the biodiversity and heterogeneity of cells, of cell fate conversions and lineage commitment, of cellular crosstalk, and of subcellular events and mechanisms that underlie cellular phenotypes. Moreover, in conjunction with other omic techniques, it demonstrated great potential in dissecting clinical observations, such as drug resistance. Single-cell omics are particularly suitable for studying highly heterogeneous and rapidly changing cell populations, such as cancer and immune cells. Recently, scRNA-seq was used on bronchoalveolar lavage fluid immune cells of COVID-19-positive patients, uncovering key immune cell populations predictive of disease severity, as well as aberrant macrophage and T cell responses in the pathogenesis COVID-19 [125]. Since proteins are the ultimate executors of cellular function, single-cell proteomic approaches are gaining momentum in promoting accurate diagnosis and prognosis, and in understanding the pathogenesis of diseases in a clinical setting [126,127,128]. In the field of cardiac research, although we have already benefitted from scRNA-seq and other single-cell omics in terms of learning the cardiac cellular complexity under physiological conditions and in major heart diseases, many underrepresented abnormalities remain to be explored. Clinically, we expect these technologies to drive the rapid identification of cellular biomarkers, enhancing the monitoring of clinical trial outcomes, predicting treatment efficacy, and supporting the development of advanced analytical methods and automated platforms in pharmaceutical research.

## Figures and Tables

**Table 1:** Summary of studies of heart development.

Study	Findings	Relevance to Heart Research
Li et al. [15] (2016)	Generated transcriptomic profiles of 2233 embryonic mouse cardiac cells using ATLAS-seq, accurately predicting the anatomical locations of individual cardiomyocytes during development.	Helps map the spatial and temporal dynamics of cardiomyocytes, aiding in understanding heart development at cellular resolution, which is crucial for dissecting lineage-traced cells like ISL1-marked cells.
DeLaughter et al. [16] (2016)	Profiled embryonic and postnatal cardiomyocytes, identifying stage-specific transcriptional programs and lineage-specific gene expression during early cardiac development.	Provides insights into the transcriptional mechanisms regulating heart development and maturation, facilitating the discovery of key genes involved in heart formation and function.
De Soysa et al. [17] (2017)	Identified Hand2 as a critical regulator of outflow tract (OFT) specification and right ventricle differentiation, using scRNA-seq to analyze cardiovascular progenitor cells (CPCs).	Offers new insights into how molecular regulators like Hand2 direct specific cardiac lineages, which is essential for understanding congenital heart defects and improving regenerative therapies.
Sereti et al. [18] (2018)	Identified Mesp1- and Nkx2-5-expressing CPCs as major sources of new cardiomyocytes, showing that α-MHC-expressing cells lose their proliferative potential after E12.5.	Contributes to understanding how cardiac progenitors contribute to heart cell populations and the timing of cardiomyocyte proliferation, which is key in cardiac regeneration research.
Xiong et al. (2019) [19]	Discovered distinct transcriptional programs for CPCs from the first and second heart fields (FHF and SHF), proposing a model of intraorgan crosstalk regulated by Nkx2-5.	Reveals the importance of cell communication in heart development and how CPCs migrate and differentiate into cardiomyocytes, providing potential therapeutic targets for heart repair.
Jia et al. [20] (2019)	Characterized CPCs marked by Nkx2-5 and Isl1, showing that Isl1 is crucial for endothelial and cardiomyocyte fate, while Nkx2-5 directs cardiomyocyte lineage.	Provides a detailed understanding of early cardiac lineage bifurcation, which is crucial for developing targeted therapies for heart diseases involving CPCs and early heart defects.
Asp et al. [21] (2020)	Created the first single-cell atlas of the human heart during early development, revealing the spatial distribution and heterogeneity of cell types at different developmental stages.	Offers a comprehensive reference for heart development, improving our ability to study congenital heart defects and develop 3D models for heart regeneration therapies.

**Table 2:** Summary of studies of adult human heart.

Study	Findings	Relevance to Heart Research
Skelly et al. [27] (2018)	Mapped intercellular communication networks in mouse hearts, identifying fibroblasts as the most communicative cells.	Highlights the critical role of fibroblasts in heart function and their response to injury, offering new therapeutic approaches for cardiac fibrosis and heart repair post-injury.
Schafer et al. [28] (2018)	Identified IL-11-positive fibroblasts as key players in fibroblast differentiation into myofibroblasts during cardiovascular disease progression.	Provides potential therapeutic targets for controlling fibrosis in cardiovascular diseases, particularly by targeting IL-11-related pathways.
Christina et al. [29] (2020)	scRNA-seq revealed that IL-6 is primarily expressed by fibroblasts and regulated by T cell-derived adenosine, indicating a purinergic metabolic interaction between fibroblasts and T cells.	Highlights the role of immune–metabolic interactions in regulating IL-6 production, a key factor in inflammation, offering potential therapeutic targets for cardiac inflammation.
See et al. [25] (2020)	Single-nucleus RNA-seq on cardiomyocytes from healthy and failing hearts uncovered heterogeneity in the transcriptomic stress response, suggesting lincRNAs as targets for cardiac repair.	Identifies long intergenic non-coding RNAs (lincRNAs) as potential therapeutic targets for promoting cardiac repair, particularly in heart failure.
Nicin et al. [30] (2020)	snRNA-seq of the cardiac septum in hypertrophic patients identified 19 distinct cell clusters, emphasizing reduced Eph receptor signaling and intercellular crosstalk in disease.	Suggests that impaired Eph receptor signaling contributes to cardiac hypertrophy, offering insights into new therapeutic strategies targeting cell communication.
Nomura et al. [32] (2019)	Unveiled the heterogeneous gene expression of hypertrophying cardiomyocytes in a mouse model of pressure overload-induced cardiac hypertrophy, identifying the role of p53 in pathogenic gene programs.	Offers insights into the molecular mechanisms driving cardiac hypertrophy, which is crucial for developing treatments to prevent or reverse heart failure.
Dick et al. [39] (2020)	Showed the heterogeneity and plasticity of cardiac macrophages, revealing that resident macrophages in the infarct area provide cardioprotective effects after myocardial infarction.	Identifies cardiac macrophages as key players in heart injury and repair, providing insights into immune-based therapies for myocardial infarction recovery.
Wehrens et al. [40] (2020)	Identified subpopulations of cardiomyocytes in hypertrophic cardiomyopathy, with one population showing greater expression of NPPA.	Suggests that targeting specific cardiomyocyte subpopulations could offer novel therapeutic approaches for treating hypertrophic cardiomyopathy and improving heart function.
Wang et al. [1] (2020)	Created the first cellular map of the adult human heart, analyzing 11,492 single cells, showing the impact of endothelial cells on cardiomyocyte function.	Highlights endothelial cells’ role in heart function and potential as therapeutic targets for heart failure.
Tucker et al. [2] (2020)	Sequenced 287,269 nuclei, identifying nine major cell types and linking them to cardiovascular diseases through genome-wide association studies.	Expands understanding of heart disease and identifies adipocytes, cardiomyocytes, and fibroblasts as therapeutic targets.
Litviňuková et al. [11] (2020)	Provided transcriptomic data on six heart regions, identifying chamber and sex-specific differences and macrophage heterogeneity.	Offers insights into cellular diversity, heart homeostasis, and new therapeutic targets based on chamber-specific cell types.
Wang et al. [41] (2021)	Revealed cardiac fibroblast subtype switching is crucial for postnatal cardiomyocyte maturation.	Identifies fibroblasts’ role in heart maturation, offering targets for regenerative therapies.
Hu et al. [42] (2017)	Found a decrease in proliferating cardiomyocytes and an increase in mature cardiomyocytes between postnatal days 6 and 10.	Key to understanding postnatal heart development, with implications for regenerative medicine.

**Table 3:** Summary of spatial transcriptomics.

Technique	Summary	Advantages	Limitations
In situ sequencing (ISS)	Directly sequences RNA within fixed tissues, preserving spatial context.	High spatial resolution; preserves tissue architecture.	Limited to fixed samples; lower throughput.
MERFISH	Uses multiplexed error-robust fluorescence in situ hybridization for RNA detection.	High sensitivity; can detect thousands of RNA species.	Complex experimental setup; data analysis can be challenging.
seqFISH	Similar to MERFISH but employs sequential hybridization of fluorescent probes.	High multiplexing capability; quantitative measurements.	Time-consuming; requires extensive optimization.
ExSeq	Extracts mRNA from single cells and sequences it with spatial information.	Maintains spatial context; suitable for low-abundance transcripts.	Lower resolution compared to other methods.
Slide-seq	Transfers RNA from tissue sections to a DNA-barcoded slide for sequencing.	High spatial resolution; can map cellular neighborhoods.	Requires specialized equipment; potential for cross-contamination.
High-definition spatial transcriptomics (HDST)	Combines tissue sectioning with sequencing to capture spatial gene expression.	High resolution; robust quantitative data.	Technical complexity; expensive.
DBiT-seq	Utilizes DNA-barcoded probes for spatial transcriptomics via digital spatial profiling.	High spatial resolution; simultaneous protein/RNA detection.	Limited number of detectable transcripts in a single run.
Stereo-seq	Combines in situ transcriptome profiling with high spatial resolution imaging.	High throughput; accurate spatial mapping of transcripts.	Requires specialized equipment; analysis can be complex.
Seq-scope	Achieves high-resolution spatial transcriptomics using a miniaturized imaging system.	High throughput; real-time imaging capabilities.	Limited detection range; requires advanced imaging systems.
PIXEL-seq	Combines high-resolution imaging with RNA sequencing, providing spatial context.	High sensitivity; allows for multiplexing.	Potential resolution constraints and the necessity for advanced imaging systems.

**Table 4:** Summary of epigenomics.

Technique	Summary	Advantages	Limitations
ATAC-seq	Identifies regions of open chromatin, which are accessible to transposase and involved in gene regulation.	High sensitivity; low cell input; rapid protocol.	Limited to accessible chromatin regions; cannot distinguish between different types of open chromatin.
ChIP-seq	Analyzes protein–DNA interactions by combining chromatin immunoprecipitation with sequencing.	Precise localization of DNA-binding proteins; works for histone modifications.	Requires high-quality antibodies; resolution depends on fragment size.
Hi-C	Captures genome-wide chromatin interactions, identifying 3D genome structure.	Provides insight into 3D genome organization; genome-wide approach.	Low resolution; difficult to resolve small chromatin interactions.
DNase-seq	Maps DNase I hypersensitive sites to locate regions of open chromatin.	Effective in detecting regulatory elements; genome-wide mapping of accessible chromatin.	Requires high-quality enzymes; may produce biased results due to DNase sensitivity.
nano-ChIP	A miniaturized version of ChIP-seq, designed for very low input or single cells.	Suitable for low cell input; maintains ChIP-seq sensitivity in small samples.	Technically challenging; requires precise handling of small samples.
iChIP	Improved ChIP-seq with higher specificity, optimized for low-input or single-cell experiments.	Higher specificity and optimized for single-cell input; reduces noise.	Complex protocol; requires optimization for low-input samples.
MOWChIP	Microfluidic ChIP for small samples with high efficiency and reduced sample loss.	Efficient for small cell populations; reduces sample loss.	Expensive; requires specialized equipment.
SurfaceChIP	Surface-enhanced ChIP using specialized surfaces for improved chromatin capture.	Improved chromatin capture; suitable for small sample sizes.	Requires specialized surfaces and techniques; high complexity.
Drop-ChIP	Single-cell ChIP sequencing technology allowing for high-throughput profiling of chromatin states.	High-throughput profiling; compatible with single-cell experiments.	Complex data analysis; requires specialized tools for single-cell data.

**Table 5:** Summary of single-cell genome and transcriptome.

Technique	Summary	Advantages	Limitations
SIDR (simultaneous isolation of gDNA and RNA)	A method for simultaneous isolation of genomic DNA (gDNA) and RNA from single cells for parallel sequencing.	Captures both genetic and transcriptomic information; preserves single-cell integrity.	Technically challenging; requires careful handling to prevent sample loss or degradation.
G&T-seq	Genome and transcriptome sequencing method for simultaneous separation and sequencing of gDNA and full-length mRNA from single cells.	Enables parallel analysis of genome and transcriptome; provides insights into gene expression and genetic variants.	Complex protocol; requires advanced sequencing tools and expertise in data integration.
DR-Seq (gDNA-mRNA sequencing)	A method that sequences both genomic DNA and mRNA from the same single cell, capturing the relationship between the genome and transcriptome.	Simultaneously provides genome and transcriptome data; useful for studying genotype–phenotype relationships.	Complex data analysis; requires optimized pipelines for both RNA and DNA sequencing data.
sci-L3-RNA/DNA	Single-cell combinatorial indexing method that allows for parallel sequencing of both RNA and DNA at the single-cell level.	High throughput; scalable to a large number of single cells; captures both RNA and DNA information.	Requires advanced bioinformatics analysis; expensive and technically complex.

**Table 6:** Summary of single-cell epigenome and transcriptome.

Technique	Summary	Advantages	Limitations
scM&T-seq	Single-cell Multiome and Transcriptome Sequencing that simultaneously profiles both genome and transcriptome from the same cell.	Captures both genetic and epigenetic information; provides multi-omic data from a single cell.	Technically challenging; requires advanced bioinformatics for data integration.
G&T-seq	Genome and transcriptome sequencing method that separates and sequences gDNA and full-length mRNA from single cells.	Enables parallel analysis of genome and transcriptome; provides insights into gene expression and genetic variants.	Complex protocol; requires advanced sequencing tools and expertise in data integration.
ScTrioseq	A method that profiles the genome, transcriptome, and DNA methylation from a single cell, capturing multi-layered molecular information.	Captures genomic, transcriptomic, and epigenomic data in a single experiment; valuable for comprehensive analysis.	Complex workflow; requires advanced bioinformatics for multi-omic data analysis.
scATAC-seq	Single-cell ATAC-seq captures open chromatin regions at single-cell resolution.	High sensitivity; low-input requirements; captures open chromatin regions at single-cell resolution.	Requires specialized equipment; complex data analysis for single-cell resolution.
scDNase-seq	Single-cell DNase I hypersensitivity sequencing maps regions of open chromatin at the single-cell level.	Effective in detecting regulatory elements at single-cell level; captures dynamic chromatin accessibility.	Technically complex; requires high-quality enzymes and optimized analysis pipelines.
scChIP-seq	Single-cell ChIP-seq identifies protein–DNA interactions or histone modifications at single-cell resolution.	Provides high-resolution insight into chromatin states and protein–DNA interactions; works for histone modifications.	Requires high-quality antibodies and optimized protocols; complex data interpretation.

In addition to me.

**Table 7:** Summary of single-cell protein expression and transcriptome.

Technique	Summary	Advantages	Limitations
SPARC	SPARC (Single-cell Profiling using RNA and PEA) combines single-cell RNA sequencing with proximity extension assay (PEA) to detect proteins and transcripts simultaneously.	Simultaneously detects RNA and protein levels; provides complementary information about gene expression and protein activity.	Technically complex; requires optimization for PEA and RNA-seq data integration.
CITE-seq	CITE-seq (Cellular Indexing of Transcriptomes and Epitopes by sequencing) combines single-cell RNA-seq with surface protein detection using DNA-barcoded antibodies.	High throughput; simultaneous measurement of mRNA and surface proteins; scalable and flexible.	Requires high-quality antibodies; limited to surface protein detection; antibody availability can be a constraint.
REAP-seq	REAP-seq (RNA Expression and Protein Sequencing) profiles both RNA and proteins at the single-cell level using antibodies conjugated with oligonucleotides.	Profiles both RNA and protein from the same cell; provides multi-modal insights into cell function.	Requires high-quality antibodies; may face challenges in data normalization between RNA and protein readouts.
ASAP-seq	ASAP-seq (Antibody Sequencing for Assessing Protein modifications) integrates single-cell transcriptomics with detection of histone modifications and surface proteins.	Integrates transcriptomics with epigenomic modifications and protein detection; provides deeper insight into chromatin states and gene expression.	Technically demanding; requires expertise in both transcriptomics and epigenomics; data integration can be challenging.
AbSeq	AbSeq (Antibody Sequencing) combines single-cell sequencing with antibody-based protein detection, allowing for simultaneous measurement of RNA and protein levels.	Simultaneous profiling of RNA and protein at single-cell resolution; helps uncover relationships between gene expression and protein function.	Dependent on antibody quality and availability; complex data analysis required to integrate protein and RNA data.

## Data Availability

No new data were created.
